# Hospital Meetings, &c.

**Published:** 1897-06-19

**Authors:** 


### HOSPITAL MEETINGS, &C.

### THE EAST FINCHLEY CONVALESCENT HOME FOR THE PARALYSED AND EPILEPTIC.

The Duchess of Albany, accompanied by Sir Robert and Lady Collins, opened the new convalescent home at East Finchley on June 16th, 1897.

For twenty-six years the National Hospital for the Paralysed and Epileptic, Queen's Square, Bloomsbury, have maintained a couple of cottages for the treatment of convalescent patients, and it celebrates the record reign by opening the above home in their stead.

The site is close by the railway station, and the new home, standing high, commands an extended and picturesque view. It is built bungalow fashion, and is nearly all on one floor. There are three wards. The largest, named after the Duchess of Albany, is for women and free; it occupies one of the wings. The "Frances" Ward, for contributing patients, is smaller, but charmingly fitted up. Next comes the dining hall, which is very handsome. One end is filled with window, some stained glass; the ceiling is gabled, with ventilator in centre, and the entrance is threefold by arches from the corridor. An effect of height is further attained by three arches, giving into the gallery above. The kitchens are exactly opposite across the corridor. The male ward, their common room, and the nurses' sitting-room, occupy the other wing.

The staircase is artistically placed, the steps are of grey marble. Upstairs are nurses and servants' quarters. Each nurse has a separate bed room, and there is a common room for their use. The corridor is continued so as to form a gallery, which gives a view of the dining hall through the arches before-mentioned. It was very useful on the opening day as the patients were enabled to see the proceedings, and it is intended that they shall use it on the occasion of any entertainment being given.

The bath-rooms and lavatories are arranged on the most modern principle, the baths being of glazed earthenware without wooden fixings, the lavatories of marble. The ventilation throughout the building has received careful attention.

The home cost £10,000 to build, and a gift from Mrs. Roget of £500 enabled it to be opened free from debt.

